# Fibroblast-Based Cell Therapy: Molecular Background, Current Therapies and Future Perspectives

**DOI:** 10.3390/cells15070613

**Published:** 2026-03-30

**Authors:** Paulina Bihuniak, Patrycja Stodolak, Piotr Kulig, Bogusław Machaliński

**Affiliations:** 1 Pharmaceutical Facility of Pomeranian Medical University, Pomeranian Medical University, 70-252 Szczecin, Poland; paulina.bihuniak@pum.edu.pl (P.B.); patrycja.stodolak@pum.edu.pl (P.S.); 2 Department of General Pathology, Pomeranian Medical University, 70-111 Szczecin, Poland; 3 Department of Hematology and Transplantology, Pomeranian Medical University, 71-252 Szczecin, Poland

**Keywords:** cell therapy, fibroblasts, ATMP, regenerative medicine, wound healing, aesthetic medicine, anti-aging medicine

## Abstract

Fibroblasts are mesenchymal cells which physiologically possess numerous functions and belong to basic cellular components necessary to maintain tissue homeostasis and are essential for extracellular matrix formation and maintenance. In addition, fibroblasts are of paramount importance in regeneration and wound healing as they interact with the immune system. These unique properties determine their great utility in cell therapies in the field of regenerative medicine. This review summarizes the mechanisms of action and clinical applications of fibroblast-based therapies as well as highlighting the future perspectives including the use of allogeneic cells.

## 1. Introduction

Fibroblasts are mesenchymal cells that constitute fundamental components of connective tissues and are essential for maintaining tissue homeostasis. They synthesize extracellular matrix (ECM) components, including collagen, elastin, glycoproteins, and glycosaminoglycans, thereby ensuring structural integrity [1]. In addition to matrix production, fibroblasts regulate ECM turnover through secretion of proteases and participation in remodeling processes [2]. Fibroblasts play a central role in regeneration and wound healing. During tissue repair, they migrate to the site of injury, contribute to matrix deposition, and may differentiate into myofibroblasts involved in wound contraction [3,4,5]. In later stages of healing, fibroblasts continue to remodel the matrix and synthesize type I collagen. Beyond structural functions, they secrete growth factors and cytokines, including FGF2, VEGF, and HGF, which regulate angiogenesis and tissue repair [3].

Fibroblasts are not merely structural cells limited to scar formation. Their pleiotropic properties are closely linked to interactions with the immune system [6]. Through autocrine and paracrine signaling, they participate in innate and adaptive immune responses and modulate recruitment and retention of leukocytes within tissues [7,8]. Stromal fibroblast populations, including fibroblastic reticular cells in lymphoid tissues, contribute to immune microenvironment organization and regulation [9,10]. Taken together, fibroblasts are involved in ECM synthesis, remodeling, regeneration, and immune regulation. These biological functions provide the basis for investigating fibroblasts as candidates for cell-based therapies in regenerative medicine. The aim of this review is to (i) summarize preclinical evidence supporting fibroblast-based therapies, (ii) evaluate clinical studies assessing their safety and efficacy, and (iii) discuss translational challenges and future directions. Despite increasing interest in fibroblast-based therapies, a comprehensive synthesis of their molecular mechanisms, clinical evidence, and translational challenges remain limited. Therefore, this review aims to provide an integrated overview of the biological basis, current clinical applications, and future perspectives of fibroblast-based cell therapy in regenerative medicine.

## 2. Search Methodology

The present review represents a narrative synthesis of available preclinical and clinical data concerning fibroblast-based cell therapies in regenerative medicine. A structured literature search was conducted using the PubMed, Scopus, and Web of Science databases covering publications from 2000 to January 2026. Search terms were applied in different combinations and included: “fibroblast cell therapy”, “autologous fibroblasts”, “allogeneic fibroblasts”, “regenerative medicine”, “wound healing”, “atrophic scars”, “tendon repair”, “dentistry”, “fibroblast heterogeneity”, and “myofibroblast differentiation”. Original experimental studies (in vitro and animal models), clinical trials (phases I–III), and translational research addressing mechanisms of action, production-related variables, safety, and therapeutic outcomes were considered eligible. In areas where higher-level evidence was limited, case reports and small case series were also considered, particularly in emerging clinical indications. Secondary sources such as narrative reviews, editorials, and conference abstracts lacking primary data were not included in the core evidence analysis, although selected publications were consulted to provide scientific context where appropriate. The identified studies were evaluated qualitatively, with particular attention to biological mechanisms, clinical indications, safety considerations, manufacturing variables, and translational challenges associated with fibroblast-based therapies.

## 3. Biological Basis of Fibroblast-Based Cell Therapy

### 3.1. Molecular Background for Fibroblast-Based Cell Therapy

Autologous fibroblasts exhibit a complex mechanism of action characterized by a wide spectrum of pleiotropic effects. Their therapeutic potential derives from the capacity to synthesize all components of the extracellular matrix (ECM) as well as from immunomodulatory properties mediated by extensive auto- and paracrine signaling. Cell culture is regarded as a substantial modification that enables classification of the cell-based product as an advanced therapy medicinal product (ATMP), during which fibroblasts acquire novel therapeutic attributes.

Through serial passaging, autologous fibroblasts progressively develop features that enhance their therapeutic efficacy in the field of regenerative medicine. Microarray-based transcriptomic profiling demonstrated that successive passages increase the proliferative potential of fibroblasts [11]. Notably, the third passage was associated with the most pronounced activation of genes involved in cell-cycle regulation and proliferation. These findings were validated by qRT-PCR, which confirmed the microarray data. Collectively, these results suggest that during in vitro expansion, fibroblasts gain the ability to sustain proliferative activity following reimplantation into recipient skin, positioning them as a promising therapeutic product for atrophic facial scar management [11].

Beyond proliferation, autologous fibroblasts acquire additional favorable characteristics relevant to scar therapy during culture. Transcriptome analysis across three sequential passages revealed that the second passage fibroblast population displayed the highest number of activated genes essential for biological skin regeneration processes, including ECM organization, collagen fibril assembly, and cell adhesion. Moreover, expression of genes encoding proteinases responsible for ECM degradation was markedly reduced at this stage [12].

Importantly, autologous fibroblasts maintain genetic stability even after multiple passages. In a murine model, transplanted fibroblasts continued to undergo mitotic divisions within recipient skin. Histopathological examination of skin biopsies collected at different time points post implantation confirmed the presence of mitotically active fibroblasts. The number of fibroblasts at implantation sites increased over time, accompanied by progressive densification of collagen fibers, in contrast to non-transplanted control areas. Crucially, no macrophage infiltration or dysplastic cell formation was observed in the grafted regions [13].

### 3.2. Fibroblast Heterogeneity and Its Significance for the Effectiveness of Cell Therapies

For a long time, scientists viewed fibroblasts as a homogeneous population of structural cells primarily responsible for the production of extracellular matrix (ECM). However, recent studies based on single-cell sequencing have shown that fibroblasts are a highly heterogeneous group of cells characterized by different phenotypic, functional, and immunological properties. These differences depend on their tissue origin, microenvironment, and proliferation history [14,15,16]. The heterogeneity of fibroblasts affects the safety and efficacy of fibroblast therapy, taking into account their regenerative potential, immunomodulatory capacity, and the risk of fibrosis and scarring.

One of the key aspects of this variability is the diversity of fibroblasts derived from different tissues, e.g., skin fibroblasts are characterized by their ability to produce type I and III collagen and repair damaged tissues. In contrast, gingival fibroblasts exhibit a more “pro-regenerative” profile, resulting in a lower tendency to fibrosis and scarring. Tendon fibroblasts, on the other hand, due to their high mechanical load, are characterized by collagen fiber production and increased expression of genes related to mechanotransduction [15,17].

At the molecular level, the heterogeneity of fibroblasts results, among other things, from different transcriptional, epigenetic, and metabolic programs that are shaped by the local tissue microenvironment. Single-cell analyses have shown that fibroblasts have numerous subpopulations with specialized functions: increased pro-inflammatory activity, strong ECM synthesis potential, and fibroblasts with immunomodulatory properties [14,16]. Some fibroblast subpopulations show a tendency to differentiate into myofibroblasts, which play a central role in fibrosis processes.

A key mechanism regulated by the microenvironment, which includes both biochemical and mechanical signals, is the differentiation of fibroblasts into myofibroblasts. Factors such as transforming growth factor β (TGF-β), tissue tension, hypoxia, and inflammatory mediators influence the expression of α-SMA and lead to the development of a myofibroblastic phenotype. This phenotype is characterized by increased contractility and enhanced ECM deposition [18,19]. This process is necessary for effective wound closure, but it can cause pathological fibrosis as well as permanent damage to the tissue structure.

The in vitro culture process is an important element in the functional heterogeneity of fibroblasts, as cell passage affects phenotypic variability and leads to a gradual loss of proliferative capacity, the appearance of cellular aging features, and changes in the expression of genes associated with ECM remodeling [20]. Some studies have shown that controlled passage can improve the phenotypic stability of cell preparations. However, there is no clear data determining what the optimal number of passages should be to clearly ensure the best compromise between production efficiency and the preservation of important regenerative properties. The lack of standardization in this regard significantly affects the comparison of research results and the evaluation of therapy effectiveness.

An important but often overlooked aspect is also the influence of donor-related factors such as age, chronic diseases, inflammation, or history of environmental exposure. These factors can affect the secretory profile of fibroblasts, their proliferation, and their susceptibility to differentiation towards myofibroblasts. All of this directly translates into the regenerative potential of autologous and allogeneic preparations [16,20].

The heterogeneity of fibroblasts takes on additional significance in the context of allogeneic fibroblast therapy. The aforementioned phenotypic diversity may increase the availability of cells with desirable properties, but on the other hand, it may cause various types of unpredictable interactions with the recipient’s immune system. Current data on the long-term survival of allogeneic fibroblasts and their immunological compatibility mainly concern animal models and small clinical trials. This significantly hinders the formulation of unambiguous, clear conclusions regarding their safety and efficacy in large patient populations.

## 4. Translational and Regulatory Considerations

Although fibroblast-based therapies are supported by a solid biological rationale and encouraging clinical safety data, several translational aspects must be clarified before these approaches can be widely integrated into routine clinical practice.

### 4.1. Dose, Administration, and Cell Persistence

Available clinical studies reveal substantial heterogeneity in administered cell dose, concentration, treatment intervals, and treated surface area [21,22,23]. Most protocols employ suspensions containing approximately 10–20 million cells/mL delivered through repeated intradermal injections. However, these regimens have not been established on the basis of formal dose–response analyses. Consequently, the relationship between injected cell number and clinical outcome remains insufficiently defined, limiting cross-study comparability and hindering the development of standardized therapeutic algorithms.

### 4.2. Manufacturing Parameters and Quality Control

Ex vivo expansion constitutes an integral component of fibroblast-based product preparation and directly influences the biological properties of the final formulation. Passage number has been shown to affect proliferative capacity and gene expression profiles in cultured dermal fibroblasts, with potential downstream effects on pathways relevant to extracellular matrix remodeling [11]. Nevertheless, an evidence-based consensus regarding the optimal passage stage that balances expansion efficiency with preservation of regenerative potency is currently lacking. For clinical application, clearly defined release criteria are required to ensure reproducibility and product safety. These typically include assessment of viability, sterility, absence of microbial contamination, and genetic stability [24]. In addition, the implementation of validated potency assays reflecting the intended mechanism of action—such as extracellular matrix synthesis or growth factor secretion—would improve comparability between studies and facilitate regulatory evaluation.

### 4.3. Regulatory Landscape

The regulatory status of fibroblast-based products depends on the extent of manipulation and intended clinical application. Within the European Union, expanded fibroblasts are generally classified as Advanced Therapy Medicinal Products (ATMPs) and are therefore subject to Good Manufacturing Practice (GMP) requirements and centralized authorization procedures. In the United States, oversight by the Food and Drug Administration (FDA) is determined by criteria such as minimal manipulation and homologous use. These jurisdiction-specific frameworks directly influence manufacturing complexity, cost structure, scalability, and ultimately the accessibility of fibroblast-based therapies.

## 5. Clinical Applications

### 5.1. Facial Contour Defects, Anti-Aging Medicine, and Atrophic Scars

As mentioned above, the injection of autologous fibroblasts in regenerative medicine has very strong preclinical rationale. In addition to preclinical studies, fibroblast-based therapy has been investigated in a clinical setting, both in experiments and clinical trials. In the first place, it was demonstrated in a case series of 3 Caucasian male donors (47, 51, and 52 years of age) that single injection of autologous cells resulted in the increase in number of fibroblasts within the dermis after cells administration. Furthermore, it was demonstrated that in the aftermath of treatment in diameter of the collagen fiber bundles occurred. The results demonstrated that administration of autologous fibroblasts may reduce age-related tissue damage [25]. Although 10-year follow-up study showed aging-related changes over the area where cells had been injected [26], these findings support the therapeutic potential of fibroblast-based treatment, as demonstrated in subsequent clinical trials.

The safety of cell-based therapies for facial contour defects has been evaluated in clinical trials. In a prospective, placebo-controlled phase III clinical trial, fibroblast injection was shown to be a safe therapeutic option for atrophic scars and other facial defects. Patients received three doses (20 million cells/mL) at 1–2 week intervals. Clinical response was sustained up to 12 months after the first administration, suggesting long-lasting effects. It should be noted that the study population was heterogeneous, including individuals of different ages, racial backgrounds, and with various facial skin disorders. Furthermore, patients in the control arm received only placebo, without any alternative standard of care (SoC) treatment [27].

In another prospective, double-blind, placebo-controlled clinical trial, the efficacy and safety of autologous fibroblasts for the treatment of atrophic acne scars on the face were investigated. Patients received up to 2 mL of autologous fibroblast suspension (10–20 million cells/mL) injected into one cheek, while the contralateral cheek was treated with placebo, at 14-day intervals. Cells were administered at a dose of approximately 0.1 mL/cm^2^. The results demonstrated that autologous fibroblast injection is a safe treatment modality for atrophic scars. The population was again highly heterogeneous, with patients of varying ages and racial origins. Importantly, the efficacy outcomes were compared against placebo rather than SoC, limiting conclusions regarding the true therapeutic superiority of fibroblasts over established methods [28].

The efficacy of autologous fibroblast transplantation was also assessed in the correction of nasolabial folds. Significant increases in total skin density (*p* = 0.0001), dermal density (*p* = 0.0001), epidermal density (*p* = 0.046), and dermal thickness (*p* = 0.036) were observed compared with baseline. Dosing was consistent with previous studies (20 million cells/mL). However, this investigation was conducted in a small cohort (*n* = 20) without a control group, again precluding conclusions regarding superiority over SoC [22].

In another study, autologous fibroblasts were shown to represent an alternative to hyaluronic acid (HA) for the treatment of nasolabial folds. Patients receiving fibroblast injections demonstrated greater therapeutic efficacy compared with the HA control arm, with outcomes at 3 months (58.41% vs. 54.67%), 6 months (52.50% vs. 46%), and 12 months (44.55% vs. 31.33%). No serious adverse events were reported [29].

Importantly, the effects of autologous fibroblast therapy appear to be long-lasting. In a non-randomized phase IIa trial conducted by Bajouri et al., favorable clinical outcomes persisted for 24 months in patients treated for wrinkles and acne scars. The study lacked a control group, using baseline status as the comparator; therefore, definitive conclusions regarding superiority over established therapies cannot be drawn [23].

Across all trials, no serious adverse events (SAEs) were reported. All adverse events were mild and resolved spontaneously within 72 h. Only transient, mild erythema was observed, likely attributable to the injection procedure itself rather than a treatment-related effect.

To date, studies have demonstrated the safety of autologous fibroblasts in the treatment of various facial skin defects. However, the design of these trials did not include an active control group. Wanitphakdeedecha et al. were the only investigators to conduct a study in which patients in the control arm were treated with hyaluronic acid for the correction of nasolabial folds [29]. Based on the currently available published data, it cannot be conclusively determined that autologous fibroblasts represent an effective treatment modality for atrophic facial scars, as treatment outcomes have not been compared against standard of care (SoC). Therefore, to definitively assess the efficacy of this intervention in the management of facial skin defects, a head-to-head clinical trial is required, directly comparing cell therapy with established therapeutic approaches, such as laser therapy for atrophic facial scars.

### 5.2. Tendon and Ligament Repair

Tendon injuries, from the epidemiological perspective, are a frequent clinical problem affecting predominantly physically active adults who are before or during their professional careers as this population is most likely to acquire trauma while engaging in sport activities. These patients frequently require surgical reconstruction of tendons and ligaments and subsequent physical therapy to achieve full recovery. From an economic perspective, therapies that facilitate faster recovery are particularly relevant, as affected individuals are often professionally active. Earlier functional restoration may reduce indirect societal costs and support return to work.

Although there are various treatment modalities for tendon and ligaments injury, which vary on the trauma mechanism, anatomical location and patients’ preference, surgical intervention followed by rehabilitation and physical therapy is in multiple clinical scenarios considered as SoC [30,31,32]. Despite advanced surgical techniques being applied, there are some challenges related to tendon repair pathophysiology to overcome. Especially, one should mention quite limited regenerative capacity due to relatively low cellularity and diminished vascularity compared to other tissues [33,34]. Fibroblasts are essential for tendon repair as they secrete all ECM components necessary to form a scar in a process resembling wound healing [34,35]. Thus, fibroblast-based cell therapy has strong pathophysiological rationale for implementation in tendon and ligament repair to enhance the long-term clinical outcomes of surgical reconstruction.

Augmentation of tendon healing through the injection of autologous fibroblasts was demonstrated to be effective after arthroscopic rotator cuff repair (ARCR) [36]. Cell application during ARCR significantly decreased the retear rate. Therefore, a fibroblast injection could be a promising biological supplement to enhance healing in these patients. Although, clinical outcomes showed no significant difference between the 2 groups at 6 months and 1 year postoperatively, results of this clinical trial provide rationale for further studies [37].

### 5.3. Wound Healing

As mentioned above, the role of fibroblasts in wound healing is paramount. Having considered the biology of tissue regeneration and scar formation, the implementation of cell therapy in this area has strong pathophysiological rationale. Indeed, several preclinical and clinical studies demonstrated the safety and feasibility of fibroblasts-based cell therapy.

Diabetic ulcers are serious complication of diabetes mellitus. Due to neuropathy as well as angiopathy, ulcers and wounds tend not to heal in this population of patients. These may lead to further complications including infections, ulcer progression, impaired mobility and a decrease in the quality of life. According to preclinical evidence autologous cells, including fibroblasts, have the potential to induce wound healing and thus fibroblast-based cell therapy appears to be a viable therapeutic strategy. For instance, Mizoguchi et al. demonstrated in an animal model (diabetic mice) that transplantation of mixed fibroblasts and peripheral blood mononuclear cells to injured sites and subsequent sealing with fibrin glue. Results demonstrated that after 9 days, wound healing was significantly more advanced at sites treated with mixed cells and sealed with fibrin than at control sites. At two weeks, wound healing further accelerated and was significantly more advanced in the same sites. Furthermore, injuries were nearly closed in all animals by 21 days. In addition, authors demonstrated that cells acted synergically with growth factors (VEGF, HGF, TGF beta) released by fibrin. This was clearly demonstrated by the fact that fibrin-mixed cell sheets have greater healing potential than cell sheets alone [37]. Moreover, the same research group further developed the method by implementing multilayer sheets and successfully tested their solution in an animal model. Once more, the study concluded that multilayered sheets promoted wound healing and microvascular angiogenesis in the skin by supplying growth factors and cytokines and thus multilayered sheets may be a promising therapeutic material for refractory cutaneous ulcers [38]. The results of another study demonstrated that the fibroblasts implantation, especially after cell modifications, could improve the healing of diabetic wound in an animal model. Furthermore, prolyl hydroxylase domain (PHD2) silenced (via siRNA) fibroblasts implantation could further improve the healing of diabetic wound. The exact mechanism was established as follows. PHD2 targeting RNA interference could elevate the level of HIF1α and VEGFa, and even increase the proliferation of fibroblasts, eventually contributing to the improvement of the wound healing in diabetic mice. It was concluded that PHD2 silenced fibroblasts implantation is an effective way to improve the diabetic wound healing [39].

Lamme et al. conducted an interesting study in which they investigated how different densities of autologous fibroblasts in dermal substitutes—either freshly seeded or precultured for 10 days—affected dermal regeneration in a full-thickness porcine wound model. Substitutes with higher numbers of fibroblast, especially the precultured high-density group, showed better cosmetic outcomes, reduced wound contraction, and fewer myofibroblasts compared to acellular controls. After six weeks, these fibroblast-rich treatments also produced less scar tissue and more mature collagen. Overall, wound healing improved proportionally to the number of fibroblasts present at transplantation [40].

The implementation of allogeneic fibroblast cell therapy was also investigated in an animal model. Morimoto et al. conducted a study in a wound animal model. After the creation of wounds an acellular collagen sponge, a collagen sponge seeded with autologous fibroblasts, and a collagen sponge seeded with allogeneic fibroblasts were transplanted. Study demonstrated the efficacy of autologous cells. Nonetheless, allogeneic fibroblasts, although survived and remained viable, did not accelerate wound healing [41].

There were also clinical reports regarding the feasibility of fibroblast-based therapy in wounds and ulcers. In a case series report of 10 diabetic patients with deep (Wagner degree 3), large, diabetic ulcers, as a coverage, meshes of in vitro expanded autologous fibroblasts were applied. Complete ulcer healing was observed in seven patients. Study concluded that the application of autologous in vitro expanded fibroblasts is a viable therapeutic option to treat large leg ulcers especially in patients with chronic diseases such as diabetes [40].

Studies investigating fibroblast-based cell therapy in regenerative medicine are summarized in Table 1.

### 5.4. Stomatology

Predictable regeneration of periodontal and peri-implant tissues remains a clinical challenge in contemporary dentistry. Tissue loss resulting from periodontal disease, trauma, or endodontic complications frequently requires complex and prolonged treatment.

Such conditions represent a significant health and economic problem, as they affect patients’ quality of life and often require prolonged therapy. Similar to the treatment of tendon and ligament injuries, the implementation of effective regenerative therapies is essential to enable patients to return more quickly to full social and professional activity.

Currently available treatment strategies include both regenerative techniques using biomaterials and conventional surgical procedures. However, the limited regenerative capacity of oral tissues has led to increasing interest in cell-based therapies. Particular attention has been given to dental pulp fibroblasts and gingival fibroblasts, which exhibit high proliferative potential and the ability to secrete extracellular matrix (ECM) components. In periodontal tissues, fibroblasts contribute to collagen deposition and modulation of local inflammatory responses.

Due to these properties, fibroblasts are considered key elements in the regenerative processes of periodontal tissues [42,43,44,45].

Dental pulp fibroblasts not only serve a structural role but also actively participate in immune responses. They can recognize inflammatory signals and initiate local defense mechanisms that promote tissue repair and regeneration [46]. The regenerative potential of these cells, particularly their ability to support tissue reconstruction, has provided the basis for research into biomimetic scaffolds that recreate the natural cellular microenvironment and promote regeneration [47]. Fibroblasts are also utilized in commercial products that combine fibroblasts and keratinocytes, applied for soft tissue augmentation and periodontal defect repair [48].

Both gingival and dental pulp fibroblasts are being investigated as potential components of regenerative strategies in dentistry.

Their ability to modulate inflammation and synthesize ECM makes them promising tools in tissue engineering and cell-based therapies. Further research on the standardization of fibroblast populations and their integration with biomaterials may, in the future, improve clinical outcomes in the treatment of periodontal diseases and the reconstruction of oral tissues. Despite promising biological properties, the available clinical evidence in dentistry remains limited to small-scale studies with heterogeneous cell expansion protocols and variable dosing strategies. Standardization of manufacturing parameters and well-designed randomized clinical trials are required to determine the precise role of fibroblast-based therapies in periodontal and peri-implant regeneration.

**Figure 1:**
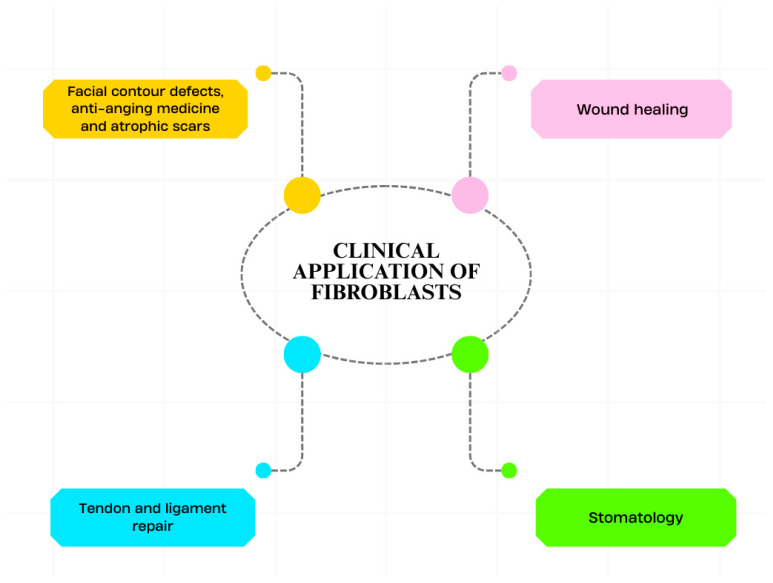
Clinical applications of fibroblast-based cell therapy. Currently, fibroblasts are implemented in many areas of regenerative medicine. Most common indications include aesthetic and anti-aging medicine, wound healing, tendon and ligament repair and stomatology.

Clinical applications of fibroblast-based cell therapy are summarized in Figure 1.

## 6. Future Perspectives

**Figure 2:**
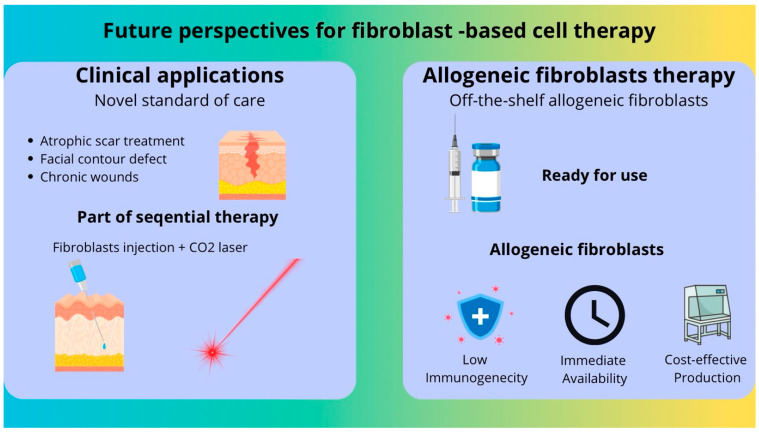
Future perspectives for fibroblast-based cell therapy.

Fibroblast-based therapy is still being developed, and novel therapeutic methods and clinical indications are to be investigated (Figure 2). In the first place, fibroblast-based therapies may potentially evolve into a component of standard therapeutic strategies in selected skin conditions, including atrophic scars, facial contour defects, chronic wounds, and burns, provided that their efficacy and long-term safety are confirmed in adequately powered randomized controlled trials.

Furthermore, in aesthetic medicine, sequential approaches combining fibroblast injections with adjunctive modalities, such as CO_2_ laser therapy, have been proposed. The rationale for such strategies is based on the hypothesis that laser-induced stimulation following cell administration may enhance local tissue remodeling processes. However, the clinical superiority of combined protocols over fibroblast therapy alone has not yet been confirmed in adequately controlled studies.

Another important consideration in the context of broader clinical implementation is the potential use of allogeneic fibroblasts. Although theoretical immunological concerns exist, preliminary preclinical and small-scale clinical data suggest acceptable short-term safety profiles in selected indications [49,50]. Nevertheless, long-term immunological compatibility and persistence of transplanted allogeneic cells require further investigation. From a translational perspective, allogeneic products offer potential advantages, including scalability of manufacturing, cost reduction, and “off-the-shelf” availability. However, their integration into routine clinical practice will depend on rigorous standardization of production protocols, well-designed randomized clinical trials, and clear regulatory pathways.

## 7. Conclusions

In conclusion, fibroblast-based cell therapy is being actively investigated across multiple clinical indications. Available preclinical and early clinical data indicate potential therapeutic relevance in facial contour defects, atrophic scars, tendon and ligament repair, chronic wounds, and selected applications in regenerative dentistry. However, variability in study design, cell expansion protocols, and dosing strategies currently limits direct comparison between studies and precludes definitive conclusions regarding clinical efficacy. Optimization of delivery platforms, including cell sheets, biomaterial scaffolds, and fibrin-based matrices, may improve therapeutic consistency. Further progress in this field will require standardized manufacturing procedures, adequately powered randomized clinical trials, and clearly defined regulatory frameworks to support safe clinical translation.

## Figures and Tables

**Table 1:** Summary of clinical studies investigating fibroblast-based cell therapy in regenerative medicine.

Indication	Study	Cell Source	Auto/Allo	N	Study Design	Dose/Regimen	Follow-Up	Main Findings/Key Limitations
Skin morphology. assessment	Machaliński et al., 2016 [25]	Postauricular skin–derived dermal fibroblasts	Autologous	3	Preliminary Study	A total of 1.5 × 10^6^ cells; 1 puncture −0.05 mL of cell suspension per 0.25 cm^2^ of skin area tested	3 months	Increased dermal fibroblast number and collagen fiber bundle diameter after cell administration; preliminary study with very small sample size.
Assessment of Extracellular Matrix Fibrous Elements	Machaliński et al., 2024 [26]	Postauricular skin–derived dermal fibroblasts	Autologous	1	Preliminary Case Study	-	10 years	Substantial changes in the organization of type I collagen within the ECM
Facial contour defects	Weiss et al., 2007 [27]	Postauricular skin–derived dermal fibroblasts	Autologous	215	Phase III, randomized, double-blind, placebo-controlled trial	20 million cells/mL; 3 injections at 1–2 week intervals	12 months	Significant improvement vs. placebo (*p* < 0.0001 at 6 months); sustained response at 12 months (81.6%); no serious adverse events. Compared to placebo only (no active comparator).
Atrophic acne scars	Munavalli et al., 2013 [28]	Postauricular skin–derived dermal fibroblasts	Autologous	99	Randomized, multicenter, double-blind, placebo-controlled trial	2 mL (10–20 million cells/mL); 3 intradermal injections at 14-day intervals	4 months	Significantly greater improvement vs. placebo (patient- and investigator-assessed); well tolerated; split-face design; no active comparator.
Nasolabial folds	Nilforoushzadeh et al., 2021 [22]	Postauricular skin–derived dermal fibroblasts	Autologous	22	Prospective, single-arm study	20 million cells/mL; 3 intradermal injections at 2-week intervals	6 months	Significant increase in dermal thickness and density (ultra sound- based); no control group; small sample size.
Nasolabial folds	Wanitphakdeedecha et al., 2023 [29]	Postauricular skin–derived dermal fibroblasts	Autologous	60	Prospective, evaluator-blinded, pilot study	3 treatments of autologous fibroblasts at 2-week intervals	12 months	Comparable efficacy to hyaluronic acid with sustained improvement; pilot study with limited sample size.
Facial contour deformities	Bajouri et al., 2020 [23]	Postauricular skin–derived dermal fibroblasts	Autologous	62	Phase IIa, open-label, single-arm study	Passage 3; 5–15 million cells/mL; 3 injections at 4–6-week intervals	24 months	Sustained clinical improvement up to 24 months; no serious adverse events; non-randomized, no control group.

## Data Availability

No new data were created or analyzed in this study.
